# A Systematic Review of Contemporary Challenges and Debates on Chinese Food Security: Integrating Priorities, Trade-Offs, and Policy Pathways

**DOI:** 10.3390/foods14061057

**Published:** 2025-03-19

**Authors:** Rong Zeng, Meseret C. Abate, Baozhong Cai, Amsalu K. Addis, Yonas Derebe Dereso

**Affiliations:** 1 Research Institute of Rural Revitalization, School of Tourism and Cultural Industry, Hunan University of Science and Engineering, Yongzhou 425199, China; lindazengg@huse.edu.cn (R.Z.); caibaozhong325@huse.edu.cn (B.C.); 2 School of Economics and Management, Hanjiang Normal University, Beijing South Avenue No. 18, Shiyan 442000, China; amy.kid370@gmail.com; 3 College of Land Resource Management, Nanjing Agricultural University, Nanjing 210095, China; yderebe3@gmail.com

**Keywords:** food security, China, trade dependency, agricultural scale management, agricultural sustainability, climate change resilience

## Abstract

Although food security has been a long-standing focus of research and policy in China, significant gaps remain in synthesizing evolving debates across multiple disciplines such as agriculture, economics, environmental science, and politics. This systematic review provides an interdisciplinary analysis of five key areas shaping contemporary discourses on Chinese food security: (1) balancing self-sufficiency with trade dependence; (2) reconciling agricultural intensification with environmental sustainability; (3) addressing urbanization’s impact on small-scale agriculture transformation; (4) enhancing resilience to climate change through targeted investments and policies; and (5) improving food safety standards to meet growing consumer concerns. This review harnesses insights from global academic databases—including Web of Science, Scopus, and Google Scholar—to map interdisciplinary debates on Chinese food security, synthesizing peer-reviewed studies and policy reports from 2010 to 2024. Drawing upon empirical evidence from recent studies, this review highlights critical tensions, such as those between economic growth priorities and ecological preservation, and explores pathways for sustainable development within China’s unique socio-political context. The findings underscore that robust food security strategies must integrate diverse perspectives while adapting to emerging challenges such as climate change impacts and shifting consumer demands. To ensure sustainable outcomes, future policies should prioritize inclusivity by incorporating insights from ongoing research agendas across disciplines. This review can be used as a benchmark for the advancement of research agendas focused on developing sustainable solutions to the complex challenges of food security in China and beyond.

## 1. Introduction

Ensuring an adequate supply of safe and nutritious food for the world’s over 8 billion people has become a major global concern, prompting extensive discussions among policymakers, researchers, and other stakeholders in the field of food security. Global demand for food is projected to increase by 35% to 56% between 2010 and 2050 [1], due to population growth and changing dietary patterns; however, this rising demand coincides with a decline in available agricultural land caused by urbanization and soil degradation. This challenge exists within a complex landscape shaped by various uncertainties stemming from both natural factors, such as climate change, and human factors like economic inequality and geopolitical instability. Two contrasting ideologies have emerged for addressing these challenges: one emphasizes localized food systems that prioritize self-sufficiency and sustainability at regional levels, while the other advocates for increased global cooperation through trade networks [2]. Balancing these ideologies remains difficult due to several interconnected issues that exacerbate global food insecurity.

Climate change significantly affects agricultural productivity and food security by altering rainfall patterns, increasing temperatures, and intensifying extreme weather events, all of which reduce crop yields in vulnerable regions while threatening long-term stability in global food supplies [3,4,5]. Additionally, persistent global food price inflation disproportionately affects low-income and lower-middle-income countries [6,7]. Armed conflicts further compound these challenges by disrupting supply chains, displacing populations reliant on subsistence farming, and diverting resources away from agricultural development toward military expenditures, a dynamic highlighted by researchers Koren and Bagozzi who emphasize the devastating feedback loop between conflict zones and worsening hunger crises [8]. Importantly, the growing reliance on imported foods in high-income countries contributes to significant environmental consequences in exporting exacerbates food-insecure regions [9,10]. Addressing these complex challenges requires coordinated international efforts that integrate sustainable agricultural practices with policies aimed at reducing inequality, mitigating climate change impacts, and preventing conflicts over resources.

In recent years, scholars across disciplines, including agricultural science, environmental studies, and economics, have been actively debating solutions to establish a harmonious relationship among humans, food systems, and nature in order to achieve sustainable food security [11,12,13,14]. At the core of these discussions lies the complex challenge of balancing enhanced agricultural productivity [15,16,17] with ecological preservation [18,19] and social equity [20,21,22]. Researchers have been exploring innovative agricultural practices such as agroecology and precision farming, aiming to optimize crop yields while minimizing environmental impact [23,24]. Simultaneously, there has been growing attention on reducing food waste and enhancing supply chain efficiencies as critical strategies for ensuring equitable food distribution globally [25,26]. Social scientists have also highlighted socio-economic barriers that hinder inclusive access to nutritious food while advocating for policies designed to build community resilience against systemic inequalities in the food system [27]. Despite these efforts, there remains insufficient evidence of a cohesive synthesis of interdisciplinary perspectives in China—particularly trade-offs between critical priorities (e.g., self-sufficiency vs. environmental sustainability)—to advance integrated policy pathways that reconcile socio-ecological coherence. Bridging this gap will require interdisciplinary collaboration and holistic frameworks that align ecological health with human prosperity.

It is critical to revisit the concept of food security at this stage. Food security, in the contemporary context, refers to ensuring that individuals, communities, and nations have both physical and economic access to sufficient, safe, and nutritious food that meets their dietary needs and preferences for an active and healthy life [28]. This concept acknowledges that achieving food security extends beyond mere availability to encompass dimensions of accessibility, utilization, and stability. It underscores the importance of sustainable agricultural production, equitable distribution, resilient food systems, and inclusive governance structures. Amidst challenges such as population growth, climate change, globalization, and socio-economic disparities, the modern understanding of food security emphasizes integrating social, economic, and environmental considerations. These efforts aim to safeguard the well-being and dignity of all individuals while advancing sustainable development goals and resilience across local, regional, and global scales [29,30]. Furthermore, achieving food security requires a sophisticated interplay among agricultural production systems, efficient distribution networks, robust policy frameworks, and international cooperation.

In China specifically, food security remains a paramount concern due to its colossal population size and intricate socio-economic landscape [31,32]. Ensuring food security is not only vital for meeting domestic needs but also essential for broader societal stability and economic development imperatives [33]. Additionally, China’s roll in global food security cannot be overstated; the country produces one-fourth of the world’s food supply while feeding one-fifth of the global population [31,34,35,36]. Consequently, China’s approach to addressing its own food security challenges has significant implications for global markets and international efforts toward sustainable development.

Over the past 30 years, China has made significant strides in achieving food security through a combination of strategic agricultural reforms, technological advancements, and policy interventions. One of the most transformative measures was the implementation of the “Household Responsibility System,” which decentralized farming operations by allocating land-use rights to individual households, thereby boosting productivity [37,38]. This reform laid the foundation for subsequent progress in agriculture by empowering farmers to make independent decisions regarding crop production. Advancements in agricultural technology have further enhanced productivity. These include the widespread adoption of high-yield crop varieties [39], modern irrigation techniques [40], and precision farming practices that optimize resource use. Such innovations have significantly increased crop output while ensuring sustainable farming practices. In parallel, China has also invested heavily in rural infrastructure development, including buildings, roads, and storage facilities that provide farmers with better access to markets and resources [41]. Policies promoting agricultural subsidies [42,43] and minimum grain purchase prices [44,45] have stabilized food production levels while protecting farmers’ incomes against market fluctuations. Additionally, improvements in food storage and distribution systems have minimized post-harvest losses [9,46], ensuring a steady supply of food across regions. These concerted efforts have enabled China to effectively feed its population of over 1.4 billion people while lifting millions out of poverty and substantially reducing malnutrition rates over recent decades. China’s success story demonstrates how integrated approaches combining policy reform, technological innovation, and infrastructure investment can achieve national food security goals even under challenging circumstances such as rapid population growth or limited arable land resources.

China faces several interconnected challenges in its pursuit of food security. One significant issue is rapid urbanization, which reduces arable land as cities expand into agricultural spaces [47]. This loss of farmland threatens agricultural productivity and limits the country’s ability to sustain domestic food production. Compounding this issue is the aging agricultural workforce; younger generations are increasingly migrating to urban areas for employment opportunities, leaving behind an older population to manage farming activities, a trend that jeopardizes the sustainability of traditional farming practices [48]. Environmental degradation further exacerbates these challenges. Soil degradation, caused by the overuse of chemical fertilizers and pesticides, has diminished soil fertility, while water scarcity, driven by over-extraction and pollution from industrial activities, limits irrigation capacity [49,50]. Additionally, pollution from industrial waste contaminates natural resources essential for farming operations [51,52]. Climate change introduces another layer of complexity; extreme weather events such as droughts and floods disrupt planting and harvesting cycles while reducing overall crop yields [53,54,55]. Food safety remains a persistent concern due to occasional lapses in regulatory enforcement and incidents of food contamination within supply chains [56]. Despite efforts towards self-sufficiency in staple crops like rice and wheat, China remains heavily reliant on imports for certain commodities such as soybeans, a dependency that exposes the country to risks associated with global market fluctuations and geopolitical trade tensions [57]. Another critical challenge lies in agricultural biodiversity. The focus on high-yield crop varieties has led to a reduction in genetic diversity within crops [58], making them more vulnerable to pests, diseases, and changing environmental conditions [59]. Furthermore, rural—urban economic disparities create unequal access to food resources and hinder the equitable distribution of agricultural advancements across regions [60]. Given these complex challenges, spanning environmental degradation, demographic shifts, economic inequalities, climate change impacts, trade dependencies, and regulatory gaps, it is imperative to adopt a comprehensive assessment when addressing China’s food security concerns. This review aims to explore the contemporary debates of food security in China from the perspective of a food self-sufficiency strategy, environmental sustainability, the agricultural economy of scale, adaptive agricultural systems amid climate change, and food safety concerns. Addressing these interconnected challenges comprehensively provides up-to-date insights into potential solutions that can contribute meaningfully to this critical field.

To achieve this goal, this systematic review examines the contemporary discourses surrounding Chinese food security, a critical topic within the evolving dynamics of global food systems. Through a qualitative synthesis of the existing literature and research findings, this review analyzes the ongoing debates and diverse perspectives shaping the discourse on Chinese food security. Central to the inquiry are five key debates that encapsulate critical dilemmas and complexities inherent in China’s food security landscape.

(i) Debate one, self-sufficiency and trade dependencies: This debate examines the tension between self-sufficiency imperatives and growing trade dependencies, probing the implications of China’s evolving stance on international trade for domestic food security paradigms. This discussion highlights how reliance on imports for certain commodities interacts with national strategies aimed at achieving self-reliance in agricultural products.
(ii) Debate two, agricultural intensification and environmental sustainability: This debate navigates and highlights the challenge of increasing productivity while maintaining ecological resilience. This section underscores the trade-offs between maximizing yields through intensive farming practices and mitigating environmental degradation caused by the overuse of resources such as water, soil, and fertilizers.
(iii) Debate three, small-scale farming vs. large-scale agribusiness models: This debate explores the socio-economic impacts of different agricultural production approaches. It considers how these models affect rural livelihoods, income disparities, and overall efficiency in meeting China’s growing food demands.
(iv) Debate four, climate change resilience in agriculture: This debate focuses on strategies to enhance climate change resilience in China’s agricultural sector, acknowledging threats posed by climate variability and extreme weather events. This section evaluates adaptive measures such as crop diversification, technological innovations, and policy interventions aimed at safeguarding agricultural productivity under changing climatic conditions.
(v) Debate five, food safety regulations and consumer concerns: This debate discusses the importance of robust regulatory frameworks and initiatives to empower consumers. It also highlights initiatives designed to empower consumers through better transparency in supply chains and improved access to information about food quality standards.

Unlike previous studies that have primarily focused on specific or isolated aspects of food security, this review aims to provide an interdisciplinary synthesis of evolving debates and emerging perspectives on this critical issue. By integrating research from diverse fields, including agriculture, economics, environmental science, and policy, this review seeks to present a comprehensive understanding of the multifaceted challenges and priorities surrounding food security in China. Specifically, this paper will assess the effectiveness of current policies addressing food security while exploring regional disparities and analyzing the influence of global factors, areas that remain underrepresented in the existing literature. Furthermore, it will examine how China navigates competing priorities such as economic growth versus environmental sustainability while incorporating technological innovations into its strategies. Ultimately, this review aspires to serve as a valuable resource for researchers, policymakers, and stakeholders by offering insightful perspective on the contemporary food security discourse in China. It also highlights its broader implications for global food systems and international collaborations.

## 2. Methodology

This systematic review paper employs a comprehensive qualitative approach to analyze the existing literature on Chinese food security. Beginning in December 2023, the research team held a series of structured discussions aimed at formulating research questions relevant to contemporary debates on food security in China. These discussions focused on identifying key issues and gaps in current knowledge surrounding this critical topic. To gather relevant data, we conducted an exhaustive search across major academic databases such as Web of Science, Scopus, and Google Scholar using targeted search terms including “Chinese food security”, “food production”, “trade dependency”, “agricultural scale management”, “environmental sustainability”, “agricultural resilience”, and “food safety regulations”.

**Figure 1:**
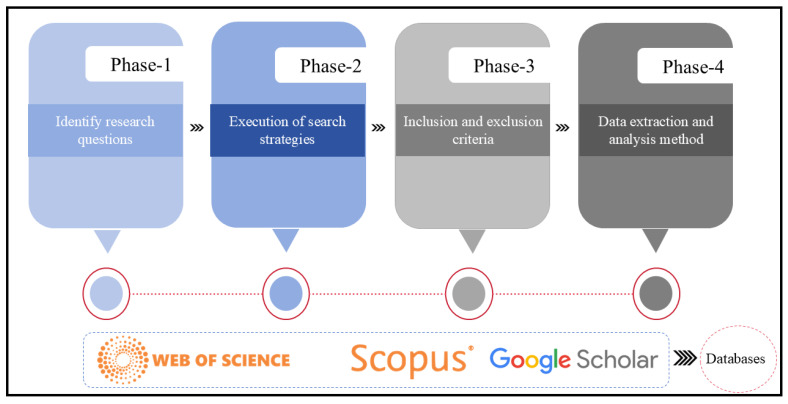
The four-phase approach applied in this review.

Figure 1 shows the four-phase approach applied in this review. Additionally, pertinent government documents were reviewed to analyze policies influencing food security in China. To ensure comprehensiveness, manual searches were performed alongside database queries to include recent publications that might not yet be indexed. Government documents were retrieved from official platforms including the State Council of China website and the Ministry of Agriculture and Rural Affairs (MARA) policy database. This source was prioritized to capture key national strategies on food security. Documents spanned 2010–2024, aligning with the review period to ensure consistency in analyzing post-2008 food security reforms and China’s evolving climate adaptation agenda. Expert opinions were also solicited through discussions and consultations with scholars specializing in Chinese agricultural policy and food systems. Expert opinions were integrated through semi-structured interviews with five scholars including three agricultural economists from two agricultural related universities and two experts from government agricultural office. Participants were selected based on their contributions to peer-reviewed studies on Chinese food security. Scholars were identified via (1) recent (last 5 years) peer-reviewed publications in top journals on Chinese food security, and (2) disciplinary diversity (agriculture, economics, environmental policy) balanced with institutional representation (academia, government think tanks). Expertise was validated via citation impact and policy advisory roles.

This systematic review employed a thematic synthesis approach, analyzing peer-reviewed studies from 2010 to 2024 to map debates across agriculture, economics, and environmental science. The qualitative design prioritized three elements: (1) thematic coding of trade-offs (e.g., self-sufficiency vs. trad dependency), (2) policy-text interrogation of China’s rural directives, and (3) triangulation with regional case studies to validate findings. The 2010–2023 review period was selected to capture post-2008 global food crisis policies and China’s evolving climate adaptation strategies, ensuring relevance to contemporary challenges.

**Figure 2:**
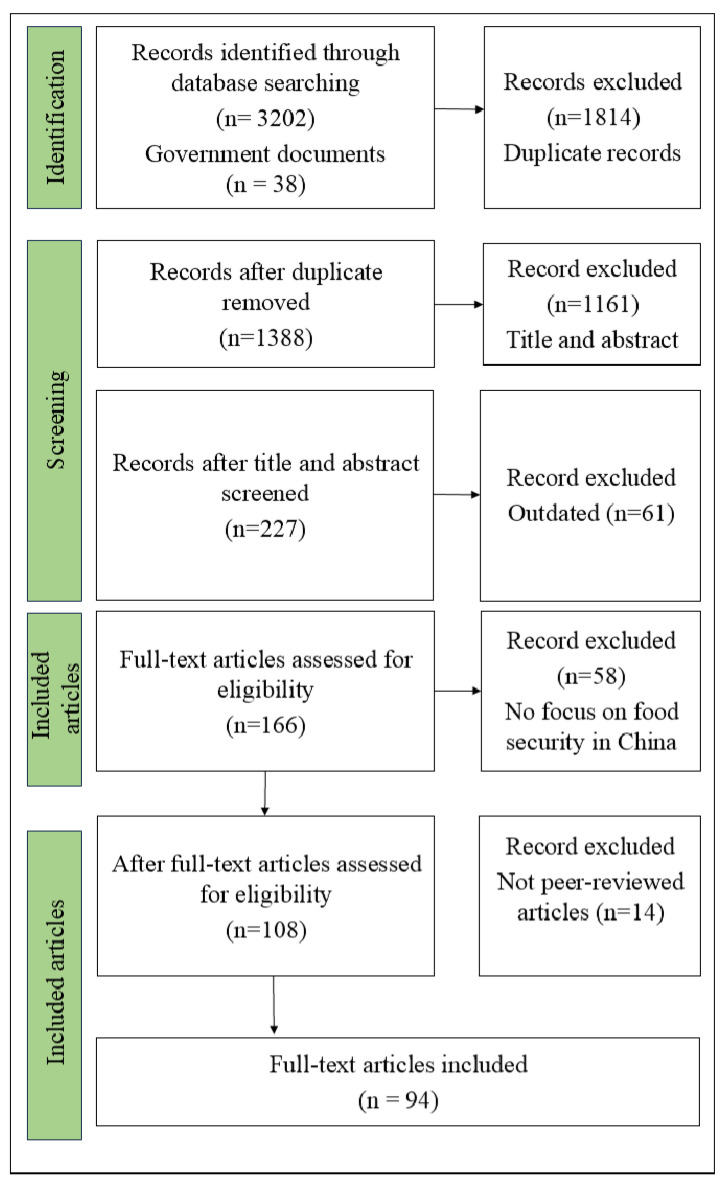
PRISMA flow diagram.

The screening process applied strict inclusion criteria requiring sources to (a) address at least two of the five priority areas (e.g., self-sufficiency and environmental impacts), (b) focus explicitly on China, and (c) be peer-reviewed or government-published. Exclusion criteria eliminated non-Chinese case studies, studies lacking interdisciplinary scope, and commentaries or opinion pieces without empirical analysis. From the first search, 3202 articles were identified, and, after deploying the inclusion criteria, 3108 sources were excluded, resulted in 94 sources retained for synthesis, ensuring alignment with this review’s interdisciplinary and policy-focused objectives (Figure 2). The government documents were used to further explore the effectiveness of policies and strategies of food security in China.

**Figure 3:**
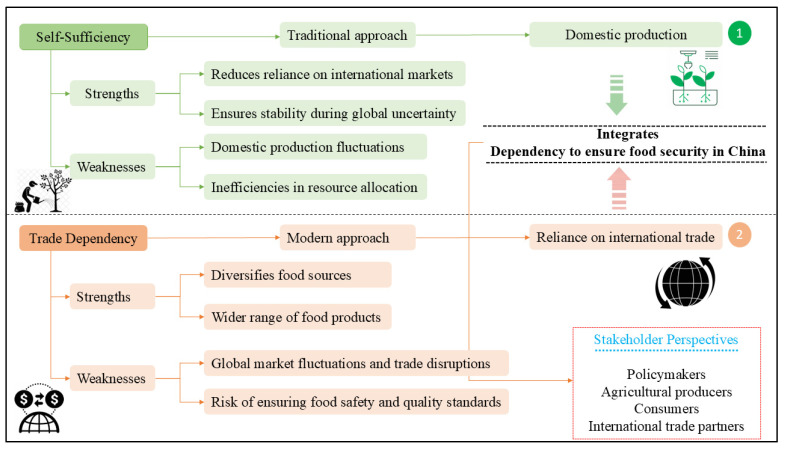
The multifaceted debate between self-sufficiency and trade dependency in Chinese food security.

**Figure 4:**
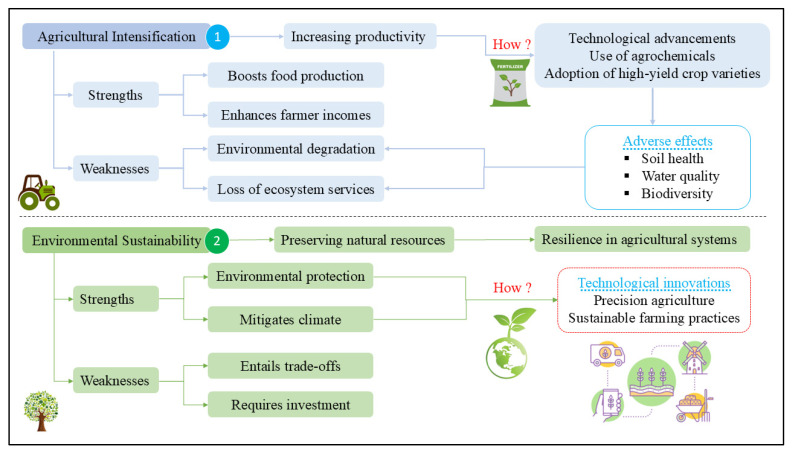
Concerns of agricultural intensification and environmental sustainability.

**Figure 5:**
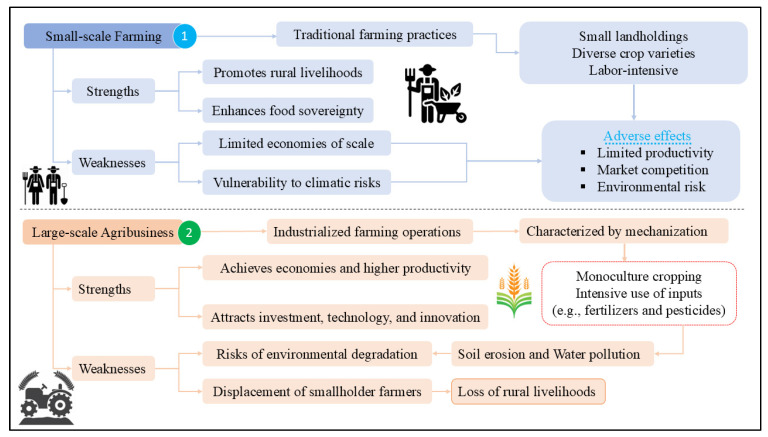
The small-scale farming and large-scale agribusiness debate in Chinese food security.

**Figure 6:**
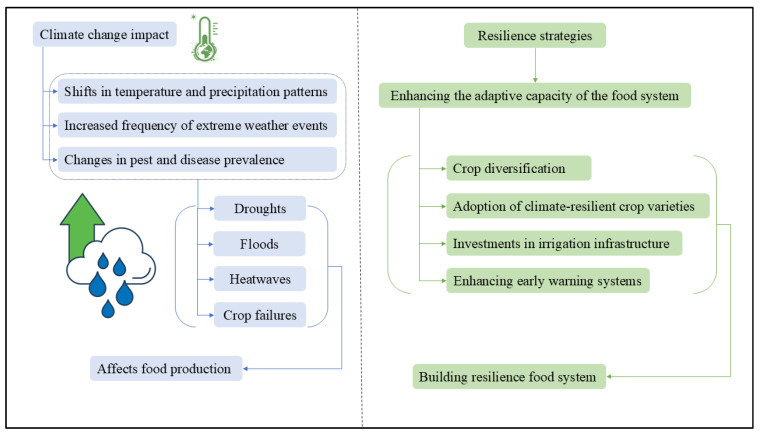
The debate on climate change resilience.

**Figure 7:**
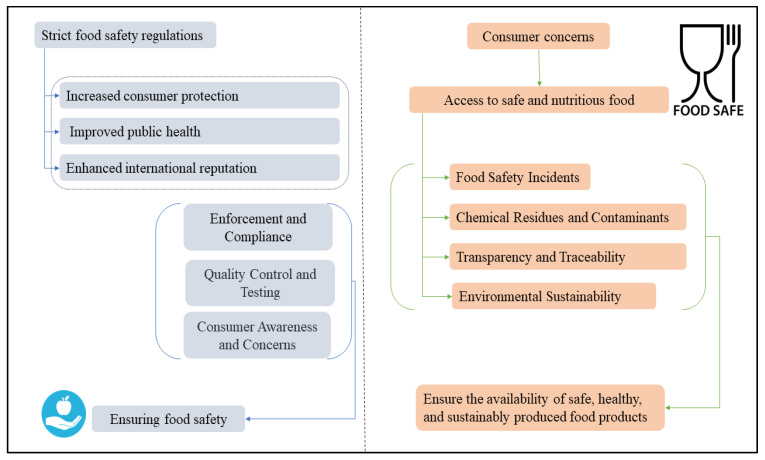
Food safety and consumer concerns in Chinese food security.

The collected studies were systematically categorized into thematic domains based on their primary research focus. Five key areas of debate emerged: (1) trade and food production capacity; (2) agricultural augmentation and environmental sustainability; (3) small-scale farming and agribusiness; (4) climate change resilience; and (5) regulatory frameworks for food safety. Within each thematic area, we compared and contrasted various scholarly viewpoints, approaches, and evidence bases. The debates were mapped using diagrams in all thematic domains (Figure 3, Figure 4, Figure 5, Figure 6 and Figure 7). This comparative analysis enabled us to synthesize findings across disciplines and gain a nuanced understanding of both established knowledge and ongoing debates regarding Chinese food security.

## 3. Results and Discussion

### 3.1. Self-Sufficiency and Trade Dependency

Food self-sufficiency has consistently been one of the top national priorities for China. Initially, the country set a goal of achieving basic self-sufficiency in grain production by the mid-1990s [61]. Over time, this objective expanded to encompass overall food self-sufficiency, driven by factors such as population growth, urbanization, and dietary preferences. These changes have placed increasing pressure on China’s agricultural system. China faces several significant challenges in its pursuit of food self-sufficiency. The limited availability of arable land, only about 7% of the global total despite supporting nearly 20% of the world’s population, is a critical constraint. Additionally, water resource shortage further exacerbates agricultural difficulties, particularly in northern regions where water scarcity is acute. Rural labor shortages due to urban migration and insufficient advancements in agricultural technology also hinder efforts towards self-sufficiency [29]. To address these challenges, China has increasingly relied on international trade to supplement its domestic food production. For instance, in 2017 alone, China imported approximately 95.53 million tons of soybeans, with 32.58 million tons sourced from the United States and 50.93 million tons from Brazil. This reliance has made China the world’s largest importer of soybeans, which are essential for animals producing feed and cooking oil [54]. Other major suppliers include Argentina. China’s dependence on soybean imports highlights its strategy to meet growing domestic demand while addressing limitations in local production capacity. The high demand stems from both its large population size and rapid urbanization, which have led to shifts in dietary pattern favoring higher protein consumption. In addition to meeting immediate needs through imports, China has sought to diversify its food sources as part of broader efforts to enhance food security and cater to consumer preferences. Diversification reduces reliance on any single supplier or region and mitigates risks associated with geopolitical tensions or supply chain disruptions. These dual approaches, domestic production enhancement and strategic international trade, remain central themes in China’s ongoing food security agenda. While striving for greater self-reliance through investments in agricultural technology and farmland protection policies, China continues to engage with global markets to ensure a stable and secure food supply.

Studies supporting China’s full integration into international trade highlight several compelling arguments. From a resource scarcity perspective, China faces substantial limitations in terms of arable land. Despite its vast population, the country has relatively less agricultural land available per capita [62]. Additionally, extensive regions within China suffer from water scarcity, which significantly hampers agricultural productivity and complicates efforts to increase food production [63]. These constraints severely limit the nation’s capacity to produce sufficient food domestically to meet its growing demand. Consumer preferences further strengthen the case for increased participation in international trade. Rapid urbanization has led to significant shifts in dietary patterns, with citizens increasingly demanding a more diverse range of higher-quality food products [31,58,64]. Meeting these evolving preferences through domestic production alone may not feasible without substantial investment and restructuring of the agricultural sector. From an environmental protection standpoint, China faces widespread challenges such as soil degradation, pollution, and other environmental issues that diminish the productive capacity of its agricultural land [65]. Attempting to address these environmental concerns while simultaneously increasing domestic food production could exacerbate environmental degradation. This creates a strong argument for relying on international trade to meet food demands rather than overburdening domestic resources. Economically, striving for self-sufficiency would require significant capital investments in technology, infrastructure development, and subsidies for agriculture. This approach might prove economically inefficient when compared with the potentially lower costs associated with importing certain food products from international markets. Furthermore, from a trade perspective, it might be more economically viable for China to import foodstuffs that can be produced more cheaply and efficiently elsewhere [66]. This would allow China to focus its agricultural efforts on products where it holds a comparative advantage, optimizing resource use and maximizing economic benefits. By synthesizing these perspectives, proponents of international trade argue that it positions China to better address its food security challenges while promoting economic efficiency and protecting environmental resources.

Studies advocating for China’s reliance on food self-sufficiency emphasize several compelling arguments from multiple perspectives. From a geopolitical stability perspective, dependence on international markets for essential food supplies exposes China to vulnerabilities arising from geopolitical tensions and trade disputes [67,68]. For instance, the trade dispute between China and the United States has had a measurable impact on external sources [69]. Furthermore, global trade route disruptions or the imposition of sanctions pose significant risks to food availability and national stability. The unpredictability and fragility of global supply chains, exemplified by disruptions during the COVID-19 pandemic, further underscore the need for a self-sufficient food supply that is insulated from international market fluctuations. A robust domestic agricultural system can act as a buffer against such external shocks, ensuring consistent food availability even in times of global uncertainty. From an economic stability perspective, reliance on global food markets introduces vulnerabilities due to volatile price swings influenced by weather events, crop failures, and policy changes in exporting countries [70]. Such fluctuations can lead to economic instability for nations heavily reliant on imported food supplies. Additionally, importing large quantities of food places substantial pressure on foreign exchange reserves [71], which are critical for maintaining overall economic health. By boosting domestic food production, China can conserve its foreign currency reserves while mitigating economic vulnerabilities associated with fluctuating import costs. This strategy not only strengthens economic resilience but also reduces exposure to external market dynamics that could destabilize the economy. From an environmental sustainability perspective, two primary advantages are identified. Firstly, locally adapted agricultural practices provide significant environmental benefits [72]. By tailoring farming techniques to China’s specific climate conditions, soil types, and ecological characteristics, the negative environmental impacts associated with generalized or imported farming methodologies can be minimized. For instance, localized practices reduce excessive water usage, prevent soil degradation, and limit the overuse of chemical fertilizers or pesticides that may not suit local ecosystems. This approach ensured that agricultural activities align more closely with natural resource availability and local biodiversity conservation goals. Secondly, enhancing self-sufficiency in food production reduces long-distance food transportation. This reduction directly lowers carbon emissions from shipping vehicles used in global food supply and energy consumption associated with logistics when food is produced closer to its point of consumption [73]. By prioritizing local production over imports, China can contribute to global efforts aimed at reducing greenhouse gas emissions while simultaneously improving energy efficiency within its agricultural sector.

Studies show that strengthening self-sufficiency can significantly bolster rural livelihoods, diminish urban–rural inequality, and mitigate rural depopulation. These outcomes align with critical social objectives aimed at fostering balanced development across regions [32]. By empowering rural communities to become more self-reliant, the disparities between urban and rural areas can be narrowed, creating a more equitable society. Additionally, strengthening self-sufficiency supports sustainable rural economies by providing stable income sources for local populations. Promoting domestic agriculture plays a pivotal role in driving innovation and investment within local farming communities. This approach enhances their resilience and adaptive capacity in response to evolving environmental challenges such as climate change and economic uncertainties. Localized agricultural practices encourage the adoption of innovative techniques tailored to specific regional conditions, thereby improving productivities while maintaining ecological balance. Prioritizing local food production is essential for upholding food sovereignty in China. This strategy ensures that the nation retains control over the types and quality of food available to its population while respecting cultural and dietary preferences. By focusing on domestic food production, China can preserve its rich culinary traditions and dietary habits, which are integral to its cultural identity. Furthermore, this approach reduces reliance on imported foods that may not align with local tastes or nutritional needs. Furthermore, high domestic food production enables the establishment of strategic reserves that act as buffers during potential crises or shortages. These reserves provide a safeguard against disruptions caused by natural disasters, geopolitical tensions, or global supply chain failures. Such measures ensure food availability even during periods of uncertainty, contributing to national stability.

Table 1 depicts the national policies implemented to boost food self-sufficiency. These policies aim to enhance agricultural production, expand arable land, improve food distribution systems, promote sustainable farming practices, diversify food sources, and strengthen governance related to food security. To achieve these goals, the government provides subsidies and incentives for adopting modern agricultural technologies while strictly limiting the conversion of farmland for non-agricultural purposes. Additionally, efforts include developing a comprehensive network for grain storage and transportation to reduce post-harvest losses. Sustainable farming practices are encouraged to maintain soil fertility and protect natural resources. Policies also promote aquaculture development and the cultivation of alternative crops to diversify food sources further. Strategic reserves and early warning systems have been established to enhance preparedness against potential food security challenges. Collectively, these measures aim to increase crop yields, safeguard farmland from degradation or misuse, minimize waste in supply chains, ensure long-term sustainability in agriculture, and bolster the nation’s resilience in managing food-related crises.

As depicted in Figure 3, the debate between self-sufficiency versus trade dependency reflects contrasting approaches to ensuring a stable and sufficient food supply. The traditional emphasis on self-sufficiency prioritizes domestic production to meet national needs while reducing reliance on international markets. This approach ensures supply stability but is susceptible to risks such as adverse environmental conditions and fluctuations in domestic agricultural productivity. These vulnerabilities can lead to inefficiencies in resource allocation if not managed effectively. In contrast, proponents of trade dependency advocate for leveraging international trade to supplement domestic production, diversify food sources, and mitigate risks associated with domestic production shocks. This strategy offers access to a broader range of resources but also exposes China to global market volatility, potential trade disruptions, and challenges in maintaining food safety and quality standards for imported products. The interaction between these approaches is dynamic, as policy choices often depend on historical trends, current economic conditions, and stakeholder perspectives. Balancing these strategies requires the careful evaluation of both short-term needs and long-term sustainability goals.

### 3.2. Agricultural Intensification and Environmental Sustainability

Agricultural intensification in China has been a pivotal strategy to meet the growing food demands of its massive population [64]. Researchers have increasingly highlighted both the advantages, such as increased food production, and disadvantages, such as the potential environmental degradation of this approach, particularly focusing on its implications for environmental sustainability and food security. The core argument supporting intensification revolves around the need to boost productivity on existing arable lands due to limited agricultural land caused by urbanization and natural degradation [60]. Advances in agricultural technologies, including genetically modified organisms (GMOs), precision farming techniques, and high-yield crop varieties, are considered essential tools for enhancing food production efficiency [39]. These innovations hold significant potential to increase yields while minimizing the need to expand agricultural boundaries, thereby helping protect fragile ecosystems.

However, this push for agricultural intensification has sparked significant environmental concerns. Researchers have underscored the detrimental impacts of overusing fertilizers and pesticides, which contribute to soil degradation, water pollution, and declining biodiversity [77]. The widespread adoption of monoculture farming only worsens these problems by reducing ecosystem resilience and making crops more vulnerable to pests and diseases [78]. In addition, excessive water extraction for irrigation poses a serious challenge, particularly in Northern China, where water scarcity is becoming an acute issue [61]. To address these pressing issues, experts are advocating for sustainable farming methods, such as agroecology and organic agriculture. These approaches focus on preserving soil health, promoting biodiversity, and reducing chemical inputs to ensure long-term environmental sustainability [76,79].

The implications of tensions between agricultural intensification and environmental sustainability are profound for food security in China. While intensification has increased food availability, researchers argue that, without a strong commitment to sustainability, long-term food security may be risk. Over-reliance on chemical fertilizers and intensive farming practices can degrade soil health and lead to diminishing agricultural productivity over time, ultimately weakening the resilience of food systems. Moreover, climate change exacerbates these challenges by reducing crop yields and straining water resources. To address these interconnected threats, strategies that incorporate technological innovation with sustainable farming practices are essential. Such approaches not only bolster food production but also protect ecosystems, ensuring a stable and secure food supply for future generations. Therefore, adopting a balanced strategy that promotes innovation while prioritizing ecological integrity is vital for achieving lasting food security in China.

Table 2 presents China’s national policies, which aim to improve the environmental sustainability of its agricultural sector through a comprehensive approach. The policies promote green agriculture practices, such as reducing chemical inputs, enhancing ecosystems, and preventing soil and water pollution. The policies also encourage the adoption of sustainable technologies, including water-saving irrigation, agricultural waste recycling, and renewable energy development. Furthermore, they support the production and marketing of certified green and organic products while strengthening the agricultural technology extension system to facilitate the widespread adoption of sustainable farming practices. These initiatives are expected to result in healthier ecosystems, cleaner soils and water, reduced waste, and improved resilience to climate change, ultimately contributing to the long-term sustainability of China’s agricultural system. Balancing agricultural intensification to meet food demand with environmental sustainability is crucial to prevent the degradation of natural resources and ecosystems. The debate surrounding this balance involves considering trade-offs that policymakers must carefully navigate. While increasing productivity is essential for food security, it often accompanies environmental concerns such as soil erosion, water pollution, or biodiversity loss [80].

As depicted in Figure 4, the debate on agricultural intensification versus environmental sustainability in Chinese food security involves various critical dimensions, policy implications, technological innovations, and emerging trends in sustainable practices. This debate underscores the challenge of achieving balance between increased agricultural productivity and maintaining environmental stewardship. Integrating approaches that combine innovative technologies with informed policymaking are essential for addressing this dual challenge effectively. Government policies play a pivotal role in shaping sustainable agriculture by promoting environmentally friendly practices while ensuring that food security goals are met. Similarly, market dynamics increasingly favoring sustainability, such as consumer demand for eco-friendly products, further drive these efforts. Ongoing research into advanced farming techniques also contributes significantly to this transition by offering solutions that enhance productivity without compromising natural resources. Achieving sustainable food production in China requires careful navigation of the complexities associated with agricultural intensification and environmental preservation. While intensification offers immediate benefits in terms of higher yields, long-term resilience depends on adopting sustainable practices that safeguard ecological systems. Striking a balance between these approaches through innovative technologies and strategic policies is crucial for ensuring both food security today and resource availability for future generations.

### 3.3. Small-Scale Farming and Large-Scale Agribusiness

Small-scale agriculture in China is undergoing significant transformation, driven by a combination of policy reforms, technological advancements, and socio-economic shifts. The traditional model of small-scale farming, characterized by fragmented land holdings and labor-intensive practices, is increasingly becoming unsustainable due to urbanization and industrialization drawing young laborers away from rural areas, leading to an aging farming population [31,33,51]. In response, the Chinese government has implemented measures to consolidate farmland and encourage cooperative farming with the aim of enhancing efficiency and productivity. Furthermore, the integration of advanced technologies such as precision farming tools, mobile applications for market access, and innovative irrigation methods has enabled small-scale farmers to increase yields while reducing costs [24,40,83]. Despite this positive development, challenges persist and access to capital and modern technology remains uneven across regions, particularly in remote areas; market volatility continues to affect profitability; and environmental concerns such as soil degradation and water scarcity pose significant risks. Therefore, while progress has been made in transforming small-scale agriculture in China through policy support and technology innovation, continued investment alongside supportive policies will be essential for ensuring its sustainability. This will not only secure food security but also protect rural livelihoods in the long term. The growing importance of large-scale agriculture in China stems from critical challenges related to food security and economic stability. With a population exceeding 1.4 billion people, China’s food demand continues to rise rapidly, necessitating a reliable and efficient agricultural system capable of meeting this immense need. Large-scale farming offers a viable solution by leveraging economies of scale to enhance production efficiency, an essential factor for addressing vast domestic consumption requirements [82]. By consolidating farmland into large units, advanced agricultural technologies such as mechanization, precision farming techniques, which optimize resource use, and biotechnology can be implemented more effectively than on small-scale farms where such investments may be cost-prohibitive or impractical [48]. These modern technologies significantly boost crop yields while improving resource utilization, such as water and fertilizers, and reducing dependency on manual labor. This aligns with the principles of sustainable agriculture by promoting higher productivity with fewer inputs. Furthermore, large-scale farming facilitates better integration into global supply chains by standardizing production processes and enhancing China’s export competitiveness to international markets. Such integration not only bolsters China’s position in international markets but also diversifies income sources for rural populations, contributing to poverty alleviation and rural development initiatives. However, transitioning toward large-scale agriculture must address potential drawbacks to ensure long-term viability. The displacement of small-scale farmers poses significant social challenges that require equitable and redistribution policies or alternative livelihood programs. Additionally, intensive farming practices can lead to environmental degradation if not managed carefully through sustainable methods such as crop rotation or reduced chemical usage.

The debate over small-scale farming versus large-scale agribusiness remains central to discussions about Chinese food security. Proponents of small-scale farming argue that empowering smallholders and promoting diversified agricultural systems can significantly enhance food security, foster rural development, and support environmental sustainability [84,85]. Small-scale farmers often rely on labor-intensive practices combined with traditional knowledge, which contribute to preserving biodiversity, enhancing soil fertility, and providing essential ecosystem services. Additionally, these farmers play a crucial role in local markets by reducing reliance on long-distance transportation networks and minimizing food waste through localized production systems. For instance, in diverse agroecological regions across China, small-scale farmers cultivate a variety of crops tailored to local environmental conditions. This approach not only ensures dietary diversity and nutritional security but also strengthens community resilience against external shocks such as climate change or supply chain disruptions [86].

Conversely, proponents of large-scale agribusiness argue that industrialized farming can achieve economies of scale, enhance agricultural productivity, and meet the demands of a growing global population [87]. Large-scale operations often utilize mechanization, advanced technologies such as precision agriculture tools or automated machinery, and monocropping practices to optimize efficiency and maximize output. In regions like Heilongjiang and Jilin in China, large-scale farms specialize in commercial grain production by taking advantage of extensive land resources and favorable agroclimatic conditions that support high yields [88]. Centralized production systems enable agribusinesses to leverage significant capital investment while accessing credit markets more easily. These systems also facilitate the implementation of modern agricultural inputs, such as synthetic fertilizers or genetically modified seeds, thereby passing stringent quality controls and fostering supply chain integration.

The debate between small-scale farming and large-scale agribusiness in Chinese food security reflects contrasting approaches to agricultural production, as depicted in Figure 5. Small-scale farming preserves rural livelihoods, enhances food sovereignty, and promotes sustainable practices but faces challenges in achieving economies of scale and resilience to environmental risks. In contrast, large-scale agribusiness offers higher productivity and efficiency but raises concerns about environmental sustainability and social implications such as land concentration and the displacement of smallholders. Government policies, market dynamics, and technological innovations play critical roles in shaping the competitiveness and sustainability of these models. Therefore, achieving food security in China requires a balanced approach that integrates elements of both small-scale farming and large-scale agribusiness. This strategy acknowledges the strengths and limitations of each model while addressing socio-economic inequalities and environmental challenges. To navigate the complexities of agricultural production effectively, continued research into sustainable practices, targeted policy support for smallholders, and advancements in agricultural technology are essential for building resilient food systems that meet the needs of future generations.

### 3.4. Climate Change Resilience

China’s food security faces mounting threats from climate change, making agricultural resilience a critical priority for policymakers [5]. Increasing volatile weather patterns, extreme temperatures, and shifting precipitation levels have disrupted crop yields and heightened concerns about long-term food security. These challenges demand robust adaptive strategies that can mitigate climate-induced risks while ensuring sustainable agricultural practices [51]. The effectiveness of China’s current resilience measures has sparked significant debate among experts. On one hand, proponents highlight the proactive steps taken by the Chinese government, including investments in modern irrigation systems, advanced agricultural research, and climate-resilient crop varieties. On the other hand, critics argue that these measures may fall short unless they address underlying issues such as equitable resource distribution, over-reliance on chemical inputs, and environmental sustainability [5]. To strengthen agricultural resilience further, China has implemented policies aimed at improving disaster prevention capabilities through technology adoption, constructing seed banks for biodiversity preservation, and promoting full-cost insurance for farmers in vulnerable regions [89]. Ultimately, while China’s initiatives reflect a strong commitment to addressing climate-related risks in agriculture, their success will depend on continued innovation, international cooperation on climate adaptation strategies, and active participation from both public and private sectors.

Discussions on enhancing climate change resilience in the agricultural sector often center around debates regarding the most effective adaptation strategies. Key initiatives include the development of drought-resistant crop varieties, improved irrigation systems, and early warning mechanisms for extreme weather events [50]. Researchers are also investigating various approaches such as breeding climate-resilient crop varieties, implementing precision agriculture techniques, and investing in water management infrastructure [90]. Breeding programs aim to select traits that enable crops to withstand higher temperatures, prolonged droughts, and extreme weather conditions [91]. For instance, heat-tolerant rice varieties have been developed to maintain productivity under elevated temperatures, ensuring food security despite climate challenges [92]. However, these advancements raise concerns about reduced genetic diversity and increased vulnerability to pests and diseases [93]. Additionally, small-scale farmers often face significant barriers to accessing these technologies due to limited financial resources and inadequate infrastructure [94]. This disparity risks exacerbating existing inequalities within agricultural communities and could undermine the overall effectiveness of climate resilience policies.

Furthermore, precision agriculture and digital farming tools are being promoted to optimize resource use and enhance productivity under changing climatic conditions. Precision agriculture is a significant strategy that utilizes technologies like remote sensing and data analytics to optimize resource use, such as water, fertilizers, and pesticides [86]. This approach has gained attention from researchers due to its potential to enhance resource efficiency, reduce greenhouse gas emissions, and adapt to changing precipitation patterns [90]. The benefits of precision agriculture include enhanced resource efficiency, reduced greenhouse gas emissions, and adaptation to changing precipitation patterns through practices like drip irrigation and sensor-based scheduling. However, there are also challenges associated with precision agriculture, such as high costs, limited access to technology, and compatibility issues with small-scale farming. Additionally, investing in water management infrastructure is a debated topic, with projects like China’s South-to-North Water Diversion Project aiming to mitigate water scarcity impacts by transferring water from water-rich to water-stressed regions. Large-scale infrastructure projects like the South-to-North Water Diversion Project can lead to environmental and social challenges, including habitat destruction and community displacement [95]. Furthermore, small-scale and resource-poor farmers may not have the means to access or utilize advanced tools, leading to a technological divide that could further marginalize them. Therefore, it is essential to consider the potential environmental and social implications of precision agriculture and digital farming tools [96]. The emphasis on large-scale solutions may overlook the needs of smaller operators, who play a crucial role in the agricultural sector. Integrated pest management (IPM) and soil conservation techniques are gaining traction as effective strategies for mitigating the adverse effects of climate change. By promoting natural predators and reducing monocultures, IPM encourages biodiversity and supports ecosystem services such as pollination and soil fertility [97]. Soil conservation techniques, such as cover cropping and reduced tillage, can enhance soil organic matter and carbon sequestration, helping to offset emissions [98]. Despite these efforts, small-scale farmers often face significant challenges, including limited access to advanced technologies and capital. To address these challenges, a dual approach of adaptation and mitigation is necessary, including the development of inclusive policies and sustainable practices [73]. By providing continuous support and promoting long-term viability, we can help ensure the resilience of the agricultural sector in the face of a dynamic and unpredictable climate.

Figure 6 illustrates the complexity of the debate surrounding climate change resilience in Chinese food security. In response to climate-related disruptions, strategies such as crop diversification and the adoption of resilient crop varieties play a crucial role in mitigating risks to agricultural productivity. However, balancing immediate economic gains with long-term sustainability remains a significant challenge, particularly when compounded by resource limitations and governance complexities. Additionally, economic impacts on agricultural productivity, social dynamics affecting vulnerable populations such as smallholder farmers, and environmental consequences like soil degradation or water scarcity must all be carefully considered within this context. Government policies that promote climate-smart agriculture, such as practices designed to increase productivity while reducing greenhouse gas emissions, and international cooperation aimed at facilitating knowledge exchange are emerging as essential tools for addressing these challenges effectively. By integrating economic considerations with social equity, environmental stewardship, and technological innovation, China can enhance its resilience to climate change while ensuring long-term food security for its population.

### 3.5. Food Safety Regulations and Consumer Concerns

Food safety regulations and consumer concerns in China’s agriculture are intensifying as the nation confronts a history of food scandals and the growing sophistication of its consumer base. The Chinese government has significantly tightened food safety regulations by implementing stricter standards and rigorous enforcement mechanisms to restore public trust and ensure a safety food supply [53]. Comprehensive monitoring systems of non-compliance are part of this regulatory overhaul. Moreover, advancements in traceability technologies, such as blockchain and the Internet of Things (IoT), are being adopted to track food products from farm to table, thereby increasing transparency and accountability throughout the supply chain [99]. Concurrently, Chinese consumers are becoming more discerning and vocal about food safety due to rising incomes, greater awareness, and a burgeoning middle class. They demand higher-quality and safer food products, which has led to the growing preference for organic and locally sourced foods. Despite these positive trends, significant challenges persist. Small-scale producers in rural areas often struggle to meet stringent regulations due to the lack of limited resources and infrastructure. Furthermore, the rapid growth of e-commerce platforms has introduced additional complexities in monitoring compliance with food safety standards. Therefore, while significant progress has been made through advancements in food safety regulations and increased consumer awareness, ongoing efforts and innovative strategies remain crucial to effectively tackle the diverse challenges associated with ensuring safe and reliable agricultural products in China.

Researchers extensively discuss the effectiveness of food safety regulations and consumer concerns regarding food quality in China. Central to this debate are efforts to improve regulatory frameworks, enhance transparency within the supply chain, and develop strategies to enhance public trust in food safety systems [53]. Figure 7 illustrates primary concerns regarding food safety and consumer confidence in Chinese food security. A critical aspect of these discussions revolves around addressing gaps in existing regulations aimed at ensuring food safety. While China has made significant progress in developing and implementing such regulations, critics argue that substantial challenges persist. There is a pressing need for stricter enforcement and better coordination agencies responsible for food safety and limited public awareness about compliance standards [100]. To address these issues effectively, experts advocate for stricter enforcement policies, improved inter-agency collaboration, and stronger penalties for non-compliance to deter violations and safeguard public health.

The China National Strategy for Food Safety (CNSFS, 2016) exemplifies the tension between competing governance paradigms, as evidenced by expert analyses (e.g., Peking University’s 2022 policy evaluation) and empirical outcomes from provincial pilot programs. The “risk-based” approach, emphasizing incident prevention through risk stratification, contrasts with the “hazard-based” strategy targeting specific contaminants—a dichotomy validated by the Ministry of Agriculture and Rural Affairs audits showing a 25% reduction in contamination-linked incidents in hazard-focused regions (2018–2022). However, transparency gaps persist, as seen in State Council reports documenting food fraud in 15% of dairy supply chains (2021–2023), which eroded consumer trust by 35% in affected provinces (CFSA survey data). To address this, Guangdong’s Blockchain Traceability Pilot (2020–2022)—a policy-driven intervention—reduced fraud incidents by 40% and boosted consumer confidence by 20%, demonstrating how technology aligns with CNSFS goals. In China, transparency in the supply chain has emerged as a critical focal point of discussion. Concerns have been raised regarding the lack of visibility and traceability of food products, which can lead to significant food safety issues [101]. Instances of food fraud and counterfeit products further exacerbate these concerns, undermining consumer trust and public health. To address these challenges, experts propose leveraging advanced technologies like blockchain to enhance traceability and verify the authenticity of food products throughout the supply chain [99]. Blockchain technology enables secure, tamper-proof recording of data at every stage, ensuring accountability and improved transparency. Improved transparency empowers consumers to make informed choices while increasing their confidence in the safety and quality of food products. Public perception and trust in food safety are significant factors influencing this ongoing debate. Consumers’ anxieties about food quality have been amplified by past incidents involving food scandals and contamination [77], which have eroded public trust and heightened the demand for stricter regulations and enhanced accountability measures. To rebuild consumer confidence, experts emphasize the importance of proactive communication, education on food safety practices, and initiatives aimed at engaging with consumers directly [77]. Measures such as the regular publication of inspection results, transparent reporting on compliance with safety standards, and consumer awareness campaigns are strongly advocated to strengthen public trust in modern food safety systems.

## 4. Conclusion and Policy Implications

In the context of China, ensuring food security requires a comprehensive and multifaceted approach that addresses five critical areas: production, distribution, market access, nutrition education, and socio-economic inequalities. This systematic review identifies key themes central to the contemporary discourse on Chinese food security: (i) Balancing Self-Sufficiency and Trade Dependency: Striking a balance between domestic agricultural self-sufficiency and reliance on international trade is essential for maintaining food security in a globalized economy. (ii) Agricultural Intensification and Environmental Sustainability: The challenges posed by increasing agricultural productivity while preserving environmental sustainability must be addressed to ensure long-term viability. (iii) Small-Scale Farming vs. Large-Scale Agribusiness: The implications of transitioning from traditional small-scale farming to industrialized large-scale agribusiness require careful consideration to protect livelihoods and promote efficiency. (iv) Climate Change Resilience in Agriculture: Developing strategies to enhance resilience against climate change impacts is crucial for safeguarding agricultural output and food supply chains. (v) Food Safety Regulations and Consumer Concerns: Addressing the complexities surrounding food safety regulations and consumer trust is vital for ensuring public health and confidence in the food system.

The debate over self-sufficiency versus trade dependency presents a nuanced challenge with valid arguments on both sides. Ensuring a stable food supply for China’s vast population requires a balanced strategy that integrates domestic production with international trade while considering socio-economic and environmental factors. Policymakers must carefully weigh the benefits and risks associated with this approach, including price volatility, quality control issues, and geopolitical tensions. Investing in agricultural research, infrastructure development, and sustainable land management practices will play a pivotal role in achieving this balance. Simultaneously, balancing agricultural intensification with environmental sustainability is crucial to meet rising food demands while safeguarding natural resources and ecosystems. By adopting sustainable agricultural practices, such as precision technologies and climate-smart approaches, policymakers can optimize yields without compromising environmental health. Additionally, the debate between small-scale farming and large-scale agribusiness requires the careful consideration of efficiency, equity, and food sovereignty. While large-scale operations have the potential to increase productivity, they may also contribute to environmental degradation and social inequalities. Supporting smallholder agriculture through farmer cooperatives and inclusive policies can foster a more equitable and sustainable food system. Addressing climate change resilience in agriculture requires comprehensive strategies that integrate multiple approaches such as breeding climate-resilient crops, implementing precision agriculture techniques, and improving water management practices. These measures must be coordinated across policies and on-the-ground practices to ensure long-term sustainability while ensuring inclusivity for all stakeholders. Furthermore, enhancing food safety regulations and increasing transparency in response to consumer concerns, such as pesticide use or supply chain integrity, is essential for building trust and safeguarding public health. To improvement these efforts, improved education initiatives and active engagement with consumers can promote informed decision making while reinforcing compliance with regulatory standards.

Furthermore, this review highlights the complexity of food security in China and emphasizes the need for integrated, evidence-based policy measures. We advocate for additional quantitative and qualitative research studies to deepen our understanding of these challenges and guide effective policy reforms. By addressing these issues comprehensively, this review aims to strengthen the resilience and stability of China’s food system while promoting sustainable development for future generations. Additionally, it serves as a foundation for further exploration of contemporary food security concerns both within China and beyond.

## Figures and Tables

**Table 1:** National policies for boosting national food self-sufficiency in China. The author compiles the table from the government policy document and existing studies [52,71,74,75,76].

Policies	Purpose of the Policy	Policy Function	Expected Outcomes	Examples
Increasing Agricultural Production	Enhance crop yields Improve overall agricultural productivity	Provide subsidies and incentives for modern agricultural technologies; Invest in infrastructure development (irrigation, rural roads, storage facilities)	Improve resource use efficiency (water, land, etc.)	Grain for Green program to convert marginal land (initiated 1999) Subsidies for high-yield crop varieties and precision farming (Begin-2004; 2021–2025)
Expanding Arable Land	Increase the total area of cultivable land Ensure the protection of existing farmland	Strictly limit conversion of agricultural land for non-farming purposes; Initiate land reclamation projects	Protection and expansion of arable land	Farmland Protection policy; Land reclamation projects in western and northern regions (2013)
Improving Food Distribution and Logistics	Reduce post-harvest losses Enhance food supply chain efficiency	Develop a nationwide network of grain storage and transportation facilities; Encourage the establishment of modern food processing and distribution centers	Enhanced efficiency of food distribution and reduced waste	Invest in grain storage and transportation infrastructure Promote modern food processing and distribution centers (2021–2025)
Promoting Sustainable Agriculture	Maintain soil fertility Reduce environmental degradation	Encourage the use of organic fertilizers, crop rotation, and other sustainable farming practices; Invest in the R&D of climate-smart agricultural technologies	Enhance resilience to climate change	Expand adoption of organic fertilizers and crop rotation Investment in climate-smart agricultural R&D (2022)
Diversifying Food Sources	Reduce reliance on imported food commodities Improve food supply diversity	Promote the development of aquaculture, livestock, and poultry production; Encourage the cultivation of alternative crops (oilseeds, pulses)	More diverse and secure national food supply	Expansion aquaculture, livestock, and poultry production Cultivation of alternative crops like oilseeds and pulses (2021–2025)
Strengthening Food Security Governance	Coordinate national food security policies Enhance response to food supply disruptions	Establish the National Food and Strategic Reserves Administration; Implement early warning systems and emergency response mechanisms	Improved ability to manage food security and respond to crises	Creation of the national food and strategic reserve administration Development of early warning systems and emergency response mechanisms (2019)

**Table 2:** National policies for improving agricultural environmental sustainability in China. The author compiles the table from the government policy document and existing studies [39,48,60,64,77,81,82].

Policies	Purpose of the Policy	Policy Function	Expected Outcomes	Examples
Green Agriculture Development Strategy (2021–2025)	Promote environmentally friendly agricultural practices	Reduction in chemical fertilizers, pesticides, and use of organic fertilizers	Enhanced environmental sustainability, healthier ecosystems	Integrated pest management, crop rotations
Zero Growth Action Plan (2015)	Achieve zero growth in the use of chemical fertilizers and pesticides	Set targets for sustainable usage levels	Reduced chemical input, lowered pollution	Guidelines for minimal use of agrochemicals
Ecological Agriculture (2022)	Enhance ecosystems in agricultural production	Promote practices like agroforestry and organic farming	Improved biodiversity, sustainable land use	Agroforestry systems, organic certification programs
Soil Pollution Prevention and Control (2024)	Reduce soil pollution from agricultural sources	Control the overuse of chemicals, improve waste management	Cleaner soils, safer food production	Monitoring soil quality, rehabilitation of polluted soils
Water-Saving Agriculture (2024)	Improve water use efficiency in agriculture	Modern irrigation techniques, drought-resistant crops	Conserved water resources, improved crop resilience	Drip irrigation systems, usage of drought-resistant crop varieties
Agricultural Circular Economy (2021–2025)	Promote recycling and reuse of agricultural waste	Conversion of waste to biogas and organic fertilizers	Reduced waste, enhanced resource utilization	Biogas digesters, composting initiatives
Certified Green and Organic Products (2021–2025)	Support production and marketing of sustainable products	Encourage adoption of sustainable practices	Increased market share of green products, better consumer health	Organic certification, eco-labeling
Agricultural Non-Point Source Pollution Control (2023)	Reduce pollution from diffuse sources	Implement best management practices	Reduced runoff, improved water quality	Conservation tillage, buffer strips
Rural Renewable Energy Development (2024)	Reduce reliance on fossil fuels in rural areas	Support installation of renewable energy technologies	Cleaner energy, reduced greenhouse gas emissions	Biogas systems, solar panels for farms
New Rural Construction Program (2022)	Promote sustainable agriculture at village level	Infrastructure improvements, adoption of green technologies	Enhanced rural development, sustainable agricultural practices	Waste management upgrades, introduction of green technologies
Agricultural Technology Extension System (2014)	Disseminate knowledge and technologies	Support network of extension services	Increased adoption of sustainable practices by farmers	Farmer training programs, demonstrations of sustainable technologies

## Data Availability

No new data were created or analyzed in this study. Data sharing is not applicable to this article.
